# Treating Depressive Symptoms in Psychosis: A Network Meta-Analysis on the Effects of Non-Verbal Therapies

**DOI:** 10.1371/journal.pone.0140637

**Published:** 2015-10-20

**Authors:** Laura A. Steenhuis, Maaike H. Nauta, Claudi L. H. Bocking, Gerdina H. M. Pijnenborg

**Affiliations:** 1 Department of Clinical Psychology and Experimental Psychopathology, Faculty of Behavioural and Social Sciences, University of Groningen, Groningen, The Netherlands; 2 Department of Clinical and Health Psychology, Faculty of Social Sciences, University of Utrecht, Utrecht, The Netherlands; 3 GGZ Drenthe, Faculty of Social Sciences, Assen, The Netherlands; Maastricht University, NETHERLANDS

## Abstract

**Aims:**

The aim of this study was to examine whether non-verbal therapies are effective in treating depressive symptoms in psychotic disorders.

**Material and Methods:**

A systematic literature search was performed in PubMed, Psychinfo, Picarta, Embase and ISI Web of Science, up to January 2015. Randomized controlled trials (RCTs) comparing a non-verbal intervention to a control condition in patients with psychotic disorders, whilst measuring depressive symptoms as a primary or secondary outcome, were included. The quality of studies was assessed using the ‘Clinical Trials Assessment Measure for psychological treatments’ (CTAM) scale. Cohen’s *d* was calculated as a measure of effect size. Using a Network Meta-analysis, both direct and indirect evidence was investigated.

**Results:**

10 RCTs were included, of which three were of high quality according to the CTAM. The direct evidence demonstrated a significant effect on the reduction in depressive symptoms relative to treatment as usual (TAU), in favor of overall non-verbal therapy (ES: -0.66, 95% C.I. = -0.88, -0.44) and music therapy (ES: -0.59, 95% C.I. = -0.85, -0.33). Combining both direct and indirect evidence, yoga therapy (ES: -0.79, 95% C.I. = -1.24, -0.35) had a significant effect on depressive symptoms, and occupational therapy (ES: 1.81, 95% C.I. = 0.81, 2.81) was less effective, relative to TAU. Exercise therapy did not show a significant effect on depressive symptoms in comparison to TAU (ES: -0.02 95% C.I. = -0.67, 0.62). Due to inconsistency of study evidence, the indirect effects should be interpreted cautiously.

**Conclusions:**

Non-verbal therapies appear to be effective in reducing depressive symptomatology in psychotic disorders, in particular music therapy and yoga therapy.

## Introduction

Depressive symptoms are common in patients with schizophrenia [1]. Depressive symptoms often fluctuate with positive symptoms [2], and partly remit when positive symptoms decrease [3]. However, evidence indicates 40% of individuals still report depressive symptoms a year after remission [4]. Depressive symptoms in psychosis are associated with poorer adherence [5], increased self-medication such as alcoholism [6], poorer outcome in terms of symptoms and daily functioning [7], and an increased risk of suicide-attempts and actual suicides [8,9]. Therefore, it is crucial to treat these symptoms adequately.

It remains unclear what the best treatment options are for depressive symptoms in people with schizophrenia. The NICE guidelines for schizophrenia [10] do not directly address the treatment of co-morbid depressive symptoms, but instead refer to the depression guidelines. Furthermore, the guidelines for depression [11] address full-blown depressive disorders but not specific concerns related to co-morbid psychosis. Depressive symptoms in psychosis are heterogeneous, may have different origins, and may take on differing levels of severity [2]. As such, guidelines for depression are likely not applicable to this group [11].

With regard to medication, current guidelines are mostly indecisive whether antidepressants should be prescribed in adjunct to antipsychotics [12]. Although there is some evidence that antidepressants may have a beneficial effect on depressive symptoms in psychosis, this evidence is based on studies with methodological weaknesses, and is therefore unconvincing [13]. In clinical practice, antipsychotics are often combined with psychosocial interventions, such as cognitive behavioral therapy for psychotic symptoms (CBTp). CBTp has moderate positive effect (Cohen’s *d*: 0.36) on mood in psychosis [14], but this is most likely a secondary effect through relief of positive symptoms. To our knowledge, no study has examined whether CBT is effective for the treatment of depressive symptoms in psychosis when targeted directly.

For major depressive disorders, various non-verbal therapies, such as music therapy [15], yoga [16], or exercise therapy [17], have demonstrated direct positive results on depressive symptoms. Non-verbal therapies of physical nature (e.g. yoga) emphasize inner physical or emotional balance, through body postures or exercising on a weekly basis. In music therapy patients are taught to express themselves by using musical interactions (e.g. singing). This therapeutic method is hypothesized to diminish severe emotional disturbances, through increasing communication, social interactions, and quality of life [18][19].

Given the indication that non-verbal therapy has a positive effect on major depressive disorders, an effect on depressive symptoms in psychotic disorders may be expected as well. A meta-analysis by Silverman in 2003 demonstrated that music therapy shows strong effects on psychotic symptoms (e.g. catatonic behavior, cognitive symptoms, general symptoms), yet the effect on depressive symptoms was not investigated [18]. In 2011, a meta-analysis by Mossler and colleagues examined the effect of music therapy in psychotic disorders [20], finding that besides an effect on general psychopathology, it also had an effect on depressive symptoms. As the aforementioned meta-analysis demonstrated this finding on the basis of two studies, the current study will update these findings with articles that have been published since then, and also include other forms of non-verbal therapy. Up to now the questions whether other forms of non-verbal therapy are an effective treatment for depressive symptoms in psychotic disorders, and which non-verbal therapy is most effective, remain unanswered.

### Aims of the study

The purpose of this study was to provide a network meta-analysis of studies on the effect of non-verbal therapies on depressive symptoms in psychotic disorders. A network meta-analysis is especially useful when there are many different existing treatments that have been compared among themselves in complex patterns of comparisons. The main aim of this study is to examine whether non-verbal therapies are an effective treatment for depressive symptoms in psychotic disorders, and as a secondary aim to examine what type of non-verbal treatment is most effective.

## Materials and Methods

### Inclusion criteria

A literature search was conducted for all randomized controlled trials evaluating the effect of a non-verbal treatment for individuals with schizophrenia. The following electronic data bases were consulted up to January 2015: PubMed, Psychinfo, Picarta, Embase and ISI Web of Science. The search strategy included the following keywords: (schizophrenia or psychotic or psychoses or psychosis) and/or (depression or ‘depressive symptoms’) and (‘non-verbal therapy’ or ‘psychomotor therapy’ or ‘physical/ aerobic exercise’ or ‘running therapy’ or yoga or ‘body-oriented therapy’ or ‘dance therapy’ or ‘art therapy’ or ‘creative therapy’).

Studies were included which utilized each of the following criteria:

- randomized controlled trials (RCTs)
- non-verbal interventions
- participants diagnosed with a psychotic disorder
- baseline and post-treatment data for depression measures.
- less than 50% missing data

Authors LAS and GHMP independently selected papers that met the inclusion criteria according to the Cochrane guidelines (PRISMA [21]): titles and abstract were screened and in case of doubt the full-text of an article was retrieved and screened using the same inclusion criteria. Moreover, a cross-reference search of the eligible articles was conducted to identify additional studies not found in the electronic search. Assessment of studies was conducted by LAS and GHMP, and in case a disagreement presented itself it was discussed until consensus was reached. Two papers were in Chinese [22][23], and a translation of necessary data and information were provided by Mossler who had reported about these studies previously [20].

### Outcomes of interest

The outcome of interest in this meta-analysis was the standardized mean change in symptoms of depression. Depression is a “state of low mood and aversion to activity that can affect a person's thoughts and feelings, behavior, and overall well-being” [24]. Depressive symptoms can be measured using depression scales for depressive disorders (e.g. the Hamilton Depression Rating Scale; [25]), depression scales for schizophrenia (e.g. the Calgary Depression Scale for Schizophrenia; [26]), depression subscales of general mental health scales (e.g. Positive and Negative Syndrome Scale, depression subscale;[27]), or specific symptoms (e.g. anhedonia) of depression (e.g. Scale for the Assessment of Negative Symptoms, Anhedonia subscale; [28]).

### Data extraction and effect size calculations

The following data were extracted:

- patient characteristics (gender, age, diagnosis, setting, duration of illness)
- intervention characteristics (types, components, duration, frequency)
- study characteristics (number of participants, blinding, randomization, control condition, drop outs, handling of missing data, outcome measures)
- means and standard deviations of depression at baseline and post-treatment.
- baseline to post-treatment correlation of treatment arms

In case the data were not available online, authors were contacted for additional information. For all outcome measurements, the standardized mean change (Cohen’s *d*) between the baseline and post-treatment assessment for each treatment arm (intervention and control groups) in the individual studies was calculated. The subsequent equation was utilized [29]:

$$\text{Cohen}\prime \text{s}\;d=\;(M_{T1}-M_{T0})/{SD}_{T0}$$

where M_T1_ is the mean of the post-treatment measure and M_T0_ is the mean of the baseline measure. SD_T0_ signifies the standard deviation of the baseline measure. The overall effect size comparing the intervention group to the control group, is calculated with the following equation:

$$Cohen\prime s\;d\;(treatment\;vs.\;control)=d_{intervention}\;-d_{control}$$

where *d*_*intervention*_ stands for the ES of the intervention arm and *d*_*control*_ for the ES of the control arm. The Standard Error (S.E.) of Cohen’s *d* was calculated, utilizing the subsequent equation [30]:

$$\text{S. E. of Chen}\prime \text{s}\;d=\frac{2(1-r)}{n}+\frac{d^{2}}{2(n-1)}$$

where *n* stands for the number of participants in the treatment arm, *r* stands for baseline to post-treatment correlation and *d* stands for effect size as calculated by Cohen’s *d*. This is calculated separately for the treatment and control group. To obtain the S.E. for the overall Cohen’s *d* treatment vs. control, one must add the S.E. from the control group to the S.E. from the intervention group and consequently compute the square root of this value.

The quality of studies was independently assessed by two raters using the ‘Clinical Trials Assessment Measure for psychological treatments’ (CTAM; [31]); a scale designed to assess the quality of psychological treatments in mental health. It assesses six aspects of trial design; the sample size and recruitment method, allocation to treatment, assessment of outcome, control groups, description of treatments, and statistical analysis. The CTAM scores can range from 0 to 100, with studies scoring above 65 considered to be of adequate quality. After individual scoring, the raters presented and discussed their scores. In case a disagreement presented itself it was discussed until consensus was reached. The scale has a good blind inter-rater agreement of 0.96 and a sufficient internal consistency with a Cronbach’s alpha of 0.691. For the sensitivity analysis, only studies scoring 65 or above on the CTAM were to be included.

### Statistical Analysis

Statistical analyses were conducted using R software, with the metafor package [32]. We used an arm-based network meta-analysis model [33] for each outcome measure, which is specifically useful when conducting a meta-analysis on RCT’s comparing many different types of treatments. This statistical method allows for direct and indirect comparisons between different treatments. A direct comparison of treatments can be made when two conditions are directly compared in a RCT. An indirect comparison is made when none of the RCTs directly compare these specific conditions, yet if trial 1 compares condition A with condition B and trial 2 compares condition A with condition C, this allows conclusions to be drawn about the effectiveness of condition B compared to condition C ([33,34]). Gathering evidence from these diverging studies creates a network of evidence, from condition A to B, from B to C and from C to A, in which all possible comparisons can be made. A network meta-analysis thus allows for the comparison of two conditions where this is very little, or even no direct comparisons. In the case where there is very little direct evidence for a comparison, indirect evidence can supplement the existing direct evidence. When there is no direct evidence for a comparison, one may choose to only use indirect evidence.

A network meta-analysis using an arm-based random-effects model was applied to estimate both direct and indirect comparisons. Based on the fitted models, a relative intervention effectiveness could be obtained for any two interventions connected with each other via the loop in the network of effect sizes. For the direct comparisons, heterogeneity was assessed using the Q statistic [35], which determines whether the variability in effect size estimates from similar studies exceeds the variation expected from sampling error. Non-significance indicates there is no significant heterogeneity and Cohen’s *d* can be interpreted reliably. For the network model, consistency in the loop of evidence was tested using z-tests [34]. Consistency in the loop of evidence in the network is vital [36], as this indicates that treatment effects of direct versus indirect comparisons of the same treatments match, allowing for accurate estimation effect sizes. Inconsistency of evidence can be the result of many different causes, such as a.) differences in participants in the different comparisons, b.) differences in the treatment (e.g. doses) or c.) different periods, settings or contexts of the studies. When evidence is consistent, the parameters are inter-related as follows:

(A−B)−(A−C)=(C−B)

When there is inconsistency in the model, the outcome of the given equation is significantly different from the stated parameter. This inconsistency was overcome by adding a dummy to the model [36]. The dummy serves to represent the discrepancy between the direct and indirect evidence from a certain comparison. Last, publication bias was examined using the Egger’s test and funnel plots [37]. There is an indication for a publication bias if the p-value of the Egger’s test is 0.1 or lower, and the funnel plots appear asymmetric.

## Results

**Fig 1:**
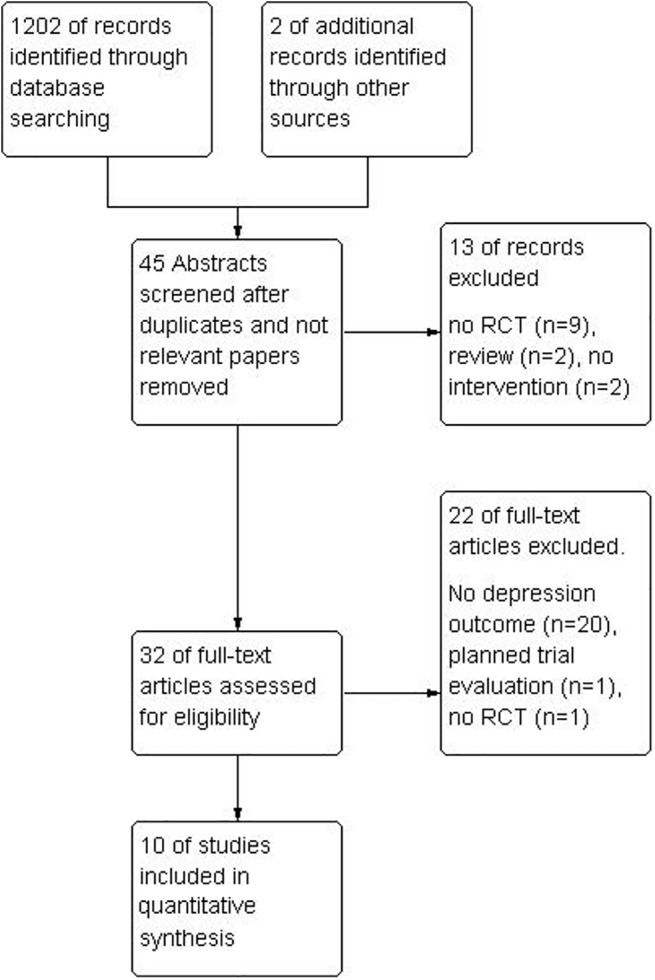
Flow diagram of the selection of studies.

The initial search resulted in 1202 records identified through database searching, with two additional records identified via cross-reference searching. Of the 1202 records, 45 abstracts were screened, which lead to an exclusion of 13 studies because the design of the study was not an RCT or the paper was a review of other studies. After a full-text screening of articles, 10 studies were included. See Fig 1 for a study flow diagram.

### Study characteristics

The search lead to the identification of 10 suitable studies for the current review. Characteristics of the included studies can be found in Table 1. The total number of participants was 576 with sample sizes ranging from 13 to 88 participants (mean = 52), consisting of 61% males (n = 350) and 39% females (n = 236). Across studies, the mean age was 40.8 (±7.5), ranging from 29.7 (±7.5) to 44.6 (±3). The studies were carried out in the following countries: Germany [38], Australia [39], U.S.A. [40], Taiwan [41], Canada [42], India [43,44][44], Netherlands [45] and China [22,23]. Interventions targeted consisted of music therapy [22,23,38,39,41], yoga therapy [40,43,44], exercise therapy [42,45], and occupational therapy [45]. Primary outcomes targeted consisted of positive symptoms [40,41,43–45], depressive symptoms [22,23], negative symptoms [38], overall physical and mental health [42], and emotional disturbance (including depression)[39].

**Table 1:** Study characteristics.

Reference	Methods	Participants*	Interventions	Outcomes^†^	CTAM score
Duraiswamy et al. (2007) [43]	Allocation: randomized, Blindness: single–assessors blinded, Duration: 4 months, Design: parallel group	Diagnosis: schizophrenia (DSM-IV), History: not reported, N = 61, Age: mean 32, range 18–55, Sex: 42 M, 19 F, Setting: inpatients and outpatients,	1. Yoga Therapy (breathing, relaxation and body posture exercises), 1 hour a day, 5 days a week for 3 weeks. N = 31. 2. Physical Therapy (brisk walking, jogging, postures and relaxation).1 hour a day, 5 days a week for 3 weeks. N = 30	Psychopathology (PANSS), Social and Occupational Functioning (SOFS), Side effects (Simpson Angus Scale for Extrapyramidal Symptoms & AIMS), Quality of Life (WHOQOL-BREF)Measurements: T0 and T1 (4 months),	67
Li et al. (2007) [22]	Allocation: randomized, Blindness: unknown, Duration: 6 weeks, Design: parallel group.	Diagnosis: schizophrenia (CCMD-3), History: not reported, N = 60. Age: mean 32, SD 12. Sex: 60 M, 0 F, Setting: inpatients	1. Receptive group music therapy (music listening, music listening in combination with verbal inductions given by the therapist, ‘positive hypnosis’/positive imagery,), 5 weekly 40-min sessions per week (total 30 sessions). N = 30. 2. Standard care (supportive counseling). N = 30.	Depression (SDS), Anxiety (SAS), Social functioning (NOSIE subscale), Measurements: T0 and T1 (6 weeks)	37
Lu et al. (2013) [41]	Allocation: randomized, Blindness: single—assessors blinded, Duration: 5 weeks, Design: parallel group.	Diagnosis: Schizophrenia (DSM-IV), History: not reported., N = 80, Age: mean: 52, Sex: 59 M, 21 F, Setting: inpatients.	1.) Music Therapy (active and passive music interventions, incl. music listening, singing, playing instruments, watching music videos and discussions). 60 min. sessions of therapy, twice a week, for 5 weeks (total 10 sessions). N = 38.2.) Treatment-as-usual (24 hour care, activities of daily living, nursing care, meal provision and social activities). N = 42.	Psychopathology (PANSS), Depression (CDSS), Measurements: T0, T1 (5 weeks) and T2 (3 months)	66
Manjunath et al. (2013) [44]	Allocation: randomized, Blindness: single—assessors blinded, Duration: 6 weeks, Design: parallel group.	Diagnosis: Schizophrenia (DSM-IV & MINI)., History: not reported., N = 88, Age: mean 31., Sex: 49 M, 39 F., Setting: inpatients	1.) Yoga therapy (breathing, relaxation, body posture), 1 hour daily sessions, first two weeks supervised, there-after instructed to practice on their own. N = 44 2.) Exercise therapy (general exercises), 1 hour daily sessions, first two weeks supervised, there-after instructed to practice on their own. N = 44.	Psychopathology (PANSS), Depression (HDRS), Severity of symptoms (CGIS), Extrapyramidal side-effects (SAS), Measurements: T0 and T1 (6 weeks)	42
Marzolini et al. (2009) [42]	Allocation: randomized, Blindness: single—assessors blinded, Duration: 12 weeks, Design: parallel group	Diagnosis: Schizophrenia/schizo-affective disorder (DSM-IV & MINI), History: not reported, N = 13, Age: mean 45, Sex: 8 M, 5 F, Setting: outpatients	1.) Exercise therapy (warm up, cardiovascular exercise sessions, resistance training). 2 times a week for 90 minutes, 12 weeks total. N = 72.) Usual care (medication). N = 6	Six minute walking test (6MWD), One repetition maximum test (1RM), Anthropometric measurements (height, body mass, resting blood pressure, and waist/hip circumference.), Adherence (attendance), Mental health inventory (MHI; subscales for anxiety, depression, behavioral control, positive affect and total score), Feedback questionnaire, Measurements: T0 and T1 (12 weeks)	38
Morgan et al. (2011) [39]	Allocation: first randomized, then quasi-randomized, Blindness: single—assessors blinded, Duration: 2 weeks, Design: parallel group	Diagnosis: schizophrenia, schizoaffective disorder or bipolar affective disorder (DSM-IV), History: not reported, N = 49, Age: mean: 36, range 17–55, Sex: 23 M, 26 F, Setting: outpatients,	1.) Music therapy (improvisation or song writing), 4 individual sessions over two weeks. Between 10–30 minutes. N = 252.) Active control (listen to a CD with relaxing sounds), 4 individual sessions over two weeks. Between 10–30 minutes. N = 24	Brief Psychiatric Rating Scale (BPRS), Calgary Interview Guide for Depression, Nurses Observation Scale for Inpatient Evaluation (NOSIE-30), Depression Anxiety Stress Scale (DASS 21), Measurements: T0, T1 (2 weeks) and T2 (6 weeks).	46
Scheewe et al. (2013) [45]	Allocation: randomized, Blindness: single—assessors blinded, Duration: 6 months, Design: parallel group	Diagnosis: schizophrenia, schizoaffective disorder or schizofreniform disorder (DSM-IV; confirmed with CASH), History: not reported, N = 63, Age: mean: 29, Sex: 46 M, 17 F, Setting: outpatients	1.) Exercise therapy (Cardiovascular exercises & muscle strength exercises), an hour of exercise twice a week for six months. N = 31.2.) Occupational therapy (creative and recreational activities, like painting or reading), N = 32.	Psychopathology (PANSS), Depression (MADRS), Need of care (CAN), Cardiorespiratory fitness (CPET), BMI, Body fat percentage (BFP), Measurements: T0 and T1 (6 months)	78
Ulrich et al. (2007) [38]	Allocation: randomized, Blindness: single—assessors blinded;assessors unaware of study aim; success of blindingverified, Duration: 4.8 weeks, Design: parallel group.	Diagnosis: schizophrenia or related psychoses (27 of 37 had F20 in ICD-10), History: not reported, N = 37, Age: mean 38, range 22–58, Sex: 20 M, 17 F, Setting: inpatients,	1. Active group music therapy (focusing on musical processes and discussion of patients’problems), on average 7.5 sessions of 60 to 105 minutes. N = 21.2. Standard care (medication, “other” activities—no detailed description given). N = 16	Mental state: SANS, Quality of life: SPG, Unable to use, Social functioning (unvalidated subscale of published scale), Satisfaction with care (unpublished scale), Measurements: T0 and T1 (5 weeks).	58
Visceglia & Lewis (2011) [40]	Allocation: randomized, Blindness: single—assessor blinded, Duration: 8 weeks, Design: parallel group.	Diagnosis: schizophrenia (DSM-IV). However, many participants had multiple diagnoses, History: not reported, N = 18, Age: mean 42 years, Sex: 12 M, 6 F, Setting: inpatients	1. Yoga therapy (breathing exercises, warm-ups, postures, and deep relaxation), 45 min twice weekly sessions for 8 weeks (total of 16 sessions). N = 10.2. Waitlist (medication—no detailed description given). N = 8	Psychopathology (PANSS), Quality of life (WHOQOL-BREF), Measurements: T0 and T1 (8 weeks)	40
Wen et al. (2005) [23]	Allocation: randomized, Blindness: unknown, Duration:6 weeks, Design: parallel group.	Diagnosis: schizophrenia (CCMD-3), History: not reported, N = 30, Age:15 to 50, Sex: 21 M, 9 F, Setting: inpatients	1. Receptive group music therapy (music listening, other music activities: dancing, discussion emphasizing the emotional aspects of the music while listening to it), five one hour sessions per week (total 30 sessions). N = 16.2. Standard care (medication only, no anxiolytic or antidepressant). N = 14	Mental state: BPRS; depression (SDS, HDRS), Unable to use—Inpatient Recovery Effect Scale (unpublished scale), Measurements: T0 and T1 (6 weeks).	32

*DSM, Diagnostic and Statistical Manual of Mental disorders; CCMD, Chinese Classification of mental disorders; MINI, The MINI-International Neuropsychiatric Interview; CASH, The Comprehensive Assessment of Symptoms and History; ICD, International Classification of Diseases; N, number; M, Males; F, Females.

^†^PANSS, Positive and Negative Syndrome Scale; SOFS, The Social and Occupational Functioning Scale, AIMS, The Abnormal Involuntary Movement Scale; WHOQOL-BREF, WHO quality of life BREF; SDS, Zung Self-Rating Depression Scale; SAS, Zung Self-Rating Anxiety Scale; NOISIE, Nurses Observation Scale for Inpatient Observation; CDSS, The Calgary Depression Scale for Schizophrenia, HDRS, Hamilton Depression Rating Scale; CGIS, The Clinical Global Impressions Scale; MHI, The Mental Health Inventory; MADRS, The Montgomery Asberg Depression Scale; CAN, Camberwell Assessment of Need; CPET, Cardiopulmonary exercise testing; SANS, Scale for the Assessment of Negative Symptoms; SPG, Scales for Mental Health; BPRS, Brief Psychiatric Rating Scale

Most studies reported baseline and post-treatment means but one study only reported the mean depression change (only 1 study; [40]). These authors [40] were contacted for the separate baseline and post-treatment means in order to make the calculation of effect sizes homogenous for all studies. None of the studies used dichotomous outcomes of depression. As only two studies reported follow-up results, namely 1 month [39] and 3 months [41] after treatment, long-term effects were not studied. Thus if studies reported multiple time-points of measurement, only those assessed immediately after treatment were used. Last, the baseline to post-treatment correlation was not reported in the individual articles and authors could not be contacted to provide it. In line with previous research [46], we assumed a conservative correlation of 0.7 for all studies. A sensitivity analysis around this correlation provided support for its use.

In the case that a study employed a measurement scale which assessed the outcome in the opposite direction (lower score indicating more depression), the effect size was multiplied by -1 (this was done for Marzolini and colleagues [42]). Moreover, in the case that a study measured the outcome variable using multiple different measurement scales [39][23], the most suitable measurement scale of depression was chosen. For the study by Morgan and colleagues [39] this was the Calgary Depression Scale for Schizophrenia (CDSS), and the study by Wen and colleagues [23], this was the Hamilton Depression Rating Scale (HDRS). This was done on the basis of a preferred hierarchy of depression scales, determined post-hoc (see Table 2). This hierarchy was based on the available evidence on the reliability and validity of the scale as a depression instrument in schizophrenia and was determined in consensus with LAS and GHMP (see: [47]). If there was no evidence available for its use in schizophrenia, general quality criteria such as reliability, specificity and validity in depressive disorder were consulted [48–52].

**Table 2:** Hierarchy of preferred depression measurement scales.

Ranking	Measurement Scale	Source for ranking
1	The Calgary Depression Scale for Schizophrenia (CDSS)	[47]
2	The Hamilton Depression Rating Scale (HDRS)	[47]
3	The Montgomery Asberg Depression Scale (MADRS)	[47]
4	Positive and negative symptom scale (PANSS—depression subscale)	[47]
5	Brief Psychiatric RatingScale (BPRS)–depression subscale	[47]
6	Depression Anxiety Stress Scale (DASS-21)	[48]
7	Zung Self-Rating Depression Scale (SDS)	[49]
8	Mental Health Inventory (Depression subscale)	[50]
9	Scale for the Assessment of Negative Symptoms (SANS—Anhedony subscale)	[51,52]

### Intervention Characteristics

The search lead to the inclusion of 10 studies examining non-verbal therapies consisting of music therapy [22,23,38,39,41], yoga therapy [40,43,44,53], exercise therapy [42,45,53], and occupational therapy [45].

Music therapy is “a systematic process of intervention wherein the therapist helps the client to promote health, using musical experiences and the relationships that develop through them as dynamic forces of change” [54]. In music therapy sessions, ortho-pedagogical techniques and a supportive way of working is often used [55]. An important focus is to stimulate social interactions and learning how to cope with problems in social settings. To achieve this, the music therapist uses the available resources of the client. Musical techniques [54] are used to structure and emphasize the playing of instruments, and the playing of an instrument is used to demonstrate that one is responsible for one's own actions (the sound stops as soon as the player stops playing). Often clients are encouraged to sing or write songs themselves. Group discussions are also used for reflecting. Generally, music therapy is given in a group setting, but one study in this meta-analysis reported an individual therapy treatment [39]. The number of sessions in the included studies ranged from 4 to 30, with an average of 17.1 sessions. The duration of the sessions lasted around 45 minutes on average, with the intervention duration lasting from 2 to 6 weeks (mean of 4.7 weeks). In the five music studies, three utilized a trained instructor [38,39,41], and two did not give enough information to determine the level of training [22,23].

Yoga is a traditional Indian approach used in complementary medicine [56]. It is suggested that yoga creates inner physical and emotional balance through the use of body postures ('asana') in combination with breathing ('pranayama'), and relaxation techniques ('nidra'). The included studies utilized an average of 15 sessions (range: 14–16), each lasting either 45 or 60 minutes. The interventions itself lasted from 2 to 8 weeks, with an average of 4.3 weeks. Two studies utilized trained yoga instructors [43,44] but one study did not note enough information regarding level of training [40]. All yoga interventions were delivered in group format.

Exercise Therapy is a relatively broad approach, which generally consists of patients engaging in regular exercises on a weekly basis. Exercise therapy can be delivered according to a protocol, consisting of cardiovascular exercises and muscle strength exercises. In the included studies, exercise sessions were led by trained psychomotor therapists. The number of sessions delivered were 24 [42] and 48 [45], lasting from 60 to 90 minutes each. Interventions lasted from 12 to 24 weeks. One of the studies delivered exercise therapy in group format [42]. Two studies utilized exercise therapy as a control group for the examination of yoga therapy [43,44]. A notable difference between exercise therapy as a control group and the intervention of interest, was that exercise therapy as a control group was delivered over a much shorter period, namely 14–15 sessions.

Occupational therapy aims to stimulate health through occupation, such as performing activities like grooming, exercising, and shopping [57]. The goal of occupational therapy is to help participants appreciate the importance of meaningful activity, and through this foster a healthy and satisfying lifestyle. Part of the therapy entails educating participants on how to manage, select, and perform activities on a daily basis [58]. In the current meta-analysis only one study looked at occupational therapy, yet as a control group for exercise therapy [45]. In this study, occupational therapy consisted of creative and recreational activities, like painting, reading, and computer activities. Sessions were led by occupational therapists, twice weekly and lasting one hour. The period of treatment was six months.

### Study Quality Assessment

The range of CTAM scores was 32 to 78 (Table 2), with only three out of ten studies considered to be of adequate quality, with a CTAM score of at least 65 [41,43,45]. Given only three studies were considered to be of high quality, we could not compute a sensitivity analysis. However, it is important to note that the three high-quality studies all reported high single effect sizes of physical therapy versus an active control condition or TAU (E.S. = -0.76 [43], -1.83 [45] and -0.69 [41]).

All studies started out with true randomization (of which two did not describe the process of randomization [22,23]), yet one study [39] became quasi-randomized throughout the trial. The reason for this alteration in design was due to the awareness of group allocation of participants. This issue was solved by allocating subjects to the treatment group for 1 month, and the following month the next subjects were assigned to the control condition. As there were no significant differences between groups on baseline measures of psychopathology, we still included the study in our meta-analysis.

Five studies note they employed independent assessors [38,39,41,43,45], with assessors being blind to group allocation in six studies [38–41,43,45]. The treatment was adequately described by all studies, with three of these employing a treatment manual [43–45]. Three studies [22,23,40] do not explicitly state the level of training of therapists. Importantly, concealment of group allocation in the randomization process was addressed by a mere two studies [41,45]. The drop-out rate ranged from 6% to 47%, with a mean dropout rate of 23%. No study had a drop-out rate higher than 50%. Two out of six studies with missing data reported a rudimentary method for investigating drop-outs (comparing baseline demographic and clinical information between the drop-outs and others; [43,44]), and two studies used intention-to-treat analysis [41,45]. When intention-to-treat data was available, this was included for the measurement of effect sizes.

### Meta-analysis: Direct comparisons

**Fig 2:**
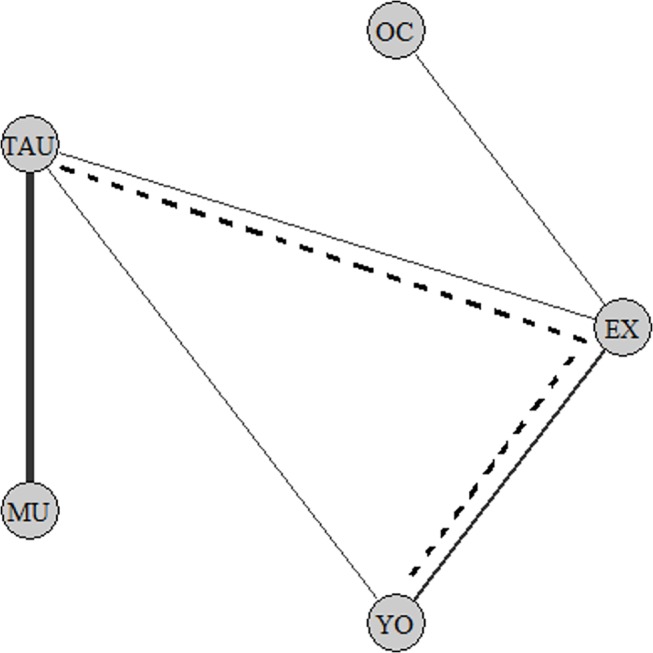
Network model of included studies. (A) MU, Music Therapy; TAU, Treatment As Usual; YO, Yoga Therapy; EX, Exercise Therapy; OC, Occupational Therapy. (B) When therapies are connected with an un-interrupting line, they have been directly compared in an RCT (for example, between YO and TAU). (C) The thickness of lines represent the number of studies that investigated this comparison. (D) An indirect comparison between YO and TAU is demonstrated by the dotted line. The comparison YO and TAU can be indirectly estimated via the loop YO to EX, from EX to TAU.

Using only direct comparisons, two comparisons can be made (see Fig 2 for a network graph of comparisons). Music versus TAU [22,23,38,39,41], or non-verbal therapy in general, covering music [22,23,38,39,41], yoga [40] and exercise [42], versus TAU. The other direct comparisons are too few in number to compute separate pooled effect sizes. To specify, there are three studies that examine yoga, but different comparison groups are used (two comparing it to exercise [43,44], and one comparing it to TAU [40]). These direct comparisons are therefore too heterogeneous to combine into one effect size.

#### (i) Music vs. TAU

**Fig 3:**
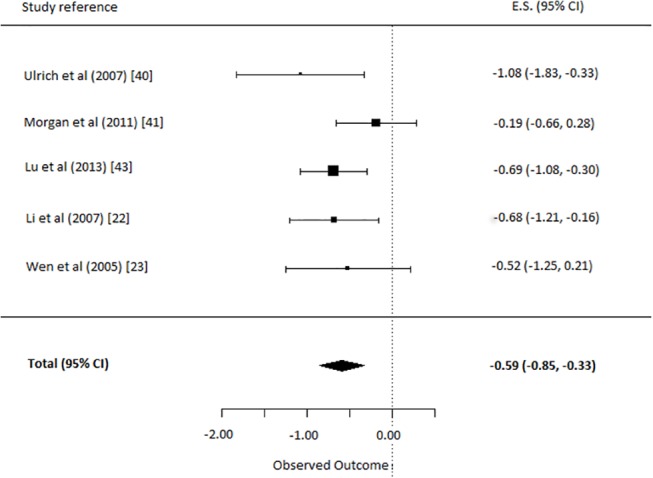
Comparison: depressive symptoms in music therapy versus treatment as usual.

For this comparison, the five studies comparing music therapy to a TAU control group were selected [22,23,38,39,41]. This yielded an overall ES of music therapy on depressive symptoms of -0.59 (95% CI = -0.85, -0.33). This was a significant and moderate effect (p<0.0001) in favor of music therapy relative to TAU (Fig 3). The q-statistic assessing heterogeneity was non-significant (*Q*(4) = 4.8, *p* = 0.31). This indicates the different music therapies had similar effects and Cohen’s *d* can be interpreted meaningfully.

#### (ii) Non-verbal therapy (Music, Yoga & Exercise) vs. TAU

**Fig 4:**
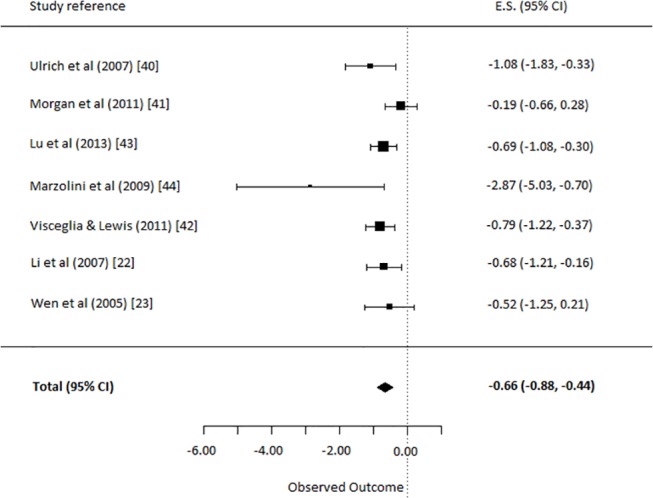
Comparison: depressive symptoms in non-verbal therapy versus treatment as usual.

For this comparison, all seven studies comparing a non-verbal therapy (Music, Yoga or Exercise) to a TAU control group were selected [22,23,38–42]. The analysis yielded an overall ES of non-verbal therapy on depressive symptoms of -0.66 (95% C.I. = -0.88, -0.44]. This was a significant and moderate to strong effect (p<0.0001) in favor of non-verbal therapy (Fig 4). The q-statistic assessing heterogeneity was non-significant (*Q*(6) = 9.54, *p* = 0.15), indicating the non-verbal therapies had similar effects and the Cohen’s *d* for this comparison can be interpreted freely.

### Network Meta-analysis: Combining Direct with Indirect comparisons

Using a network meta-analysis, the loop in the network model can be utilized to determine the different effect sizes for all treatments (see Fig 2). For example, even though there is only one study directly investigating yoga versus TAU (yoga–TAU) [40], this comparison can now additionally be estimated using the indirect comparisons of yoga versus exercise (yoga–exercise) [43,44], to exercise versus TAU (exercise–TAU) [42]. Therefore, the direct evidence will be supplemented with indirect evidence. This yields more evidence for a certain comparison, thus allowing for separate effect sizes of all treatments to be calculated separately.

#### Checking consistency

Inconsistency was tested using the following equation:

(Yoga−TAU)−(Yoga−Exercise)=(Exercise−TAU)

The z-test revealed that the outcome of the given equation is significantly different from the stated parameter (*z* = 2.47, *p* = 0.01), indicating the evidence in the loop is not consistent.

#### Source of Inconsistency

**Fig 5:**
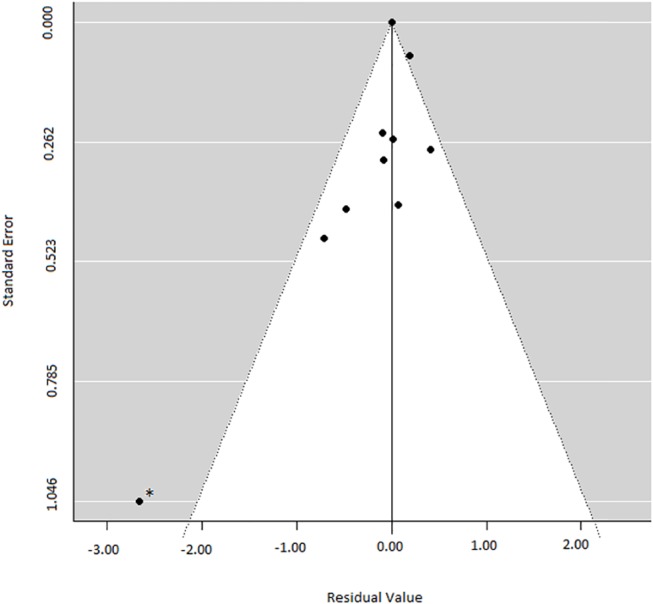
Funnel plot of included studies. * Potential outlier. Study by Marzolini et al (2009) [42].

On the basis of visual inspection of the individual effect sizes and funnel plots (see Fig 5) it appears the comparison between exercise and TAU from Marzolini and colleagues [42] is a potential outlier in the data (E.S. -2.87). Reasons could be that it was the only study that delivered treatment in group format, or that the study has a very small sample size. A dummy was added to represent the discrepancy between the direct and indirect evidence from the Exercise to TAU comparison. Therefore all coefficients in the model now exclude the direct evidence that differs from the indirect evidence, in the Exercise to TAU comparison. This causes some coefficients to be based only on indirect evidence or direct evidence, which should be kept in mind when interpreting the data.

#### Music, Yoga, Exercise & Occupational therapy vs. TAU

**Fig 6:**
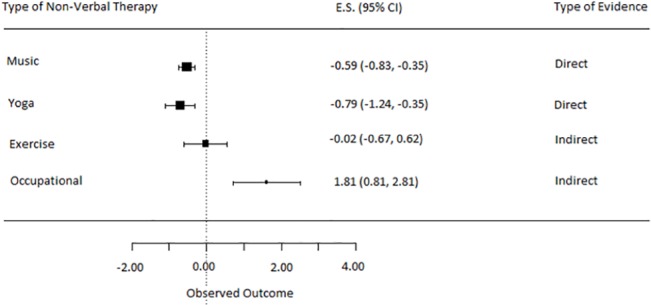
Network comparisons on depressive symptoms of all non-verbal therapies versus treatment as usual.

For the network meta-analysis all ten studies were included, which yields a separate effect size for all non-verbal treatments. This was done using both direct and indirect evidence (for effect sizes and utilized evidence: see Fig 6). The overall ES of music therapy on depressive symptoms was -0.59 (95% CI = -0.83, -0.35). This was a significant and moderate effect (p<0.0001) in favor of music therapy relative to TAU. The overall ES of yoga on depressive symptoms was -0.79 (95% CI = -1.24, -0.35). It is a significant and strong effect (p<0.0001) in favor of yoga therapy relative to TAU. The overall ES of exercise on depressive symptoms was -0.02 (95% CI = -0.67, 0.62), which is not significant. The overall ES of occupational therapy on depressive symptoms was 1.81 (95% CI = 0.81, 2.81). This was a significant and strong effect (p<0.0001) in favor of TAU relative to occupational therapy. Based on this evidence, yoga therapy appeared the most effective at reducing depressive symptomatology.

### Publication bias

The egger’s test for small-study effects found no indication for a publication bias for the direct music therapy comparison (p =. 52), but for the direct non-verbal therapy comparison (p = .06) a publication bias could not be ruled out. This indicates that the direct non-verbal therapy estimate may be inflated. The funnel plot (Fig 5) demonstrates one potential outlier, namely the study by Marzolini and colleagues [36]. This is also the study which potentially created inconsistency in our network model. We also computed the egger’s test for our network model, finding no indication for a publication bias (p = .28), but note that this network model already corrected for the outlier. The uncorrected network model also demonstrates a publication bias with the egger’s test (p = 0.01), again likely due to this one outlier.

## Discussion

The present study was conducted to investigate whether non-verbal therapy is an effective treatment for depressive symptoms in psychotic disorders. In addition, we attempted to examine which non-verbal therapy was most effective. Through a systematic search we identified ten studies, of which five investigated music therapy, three investigated yoga therapy, and two investigated exercise therapy (three were of high quality according to the CTAM). When only examining direct comparisons using regular meta-analytic techniques, our findings show a strong effect of overall non-verbal therapy, and a moderate effect of specifically music therapy on the reduction of depressive symptomatology. Using a network meta-analysis, we were able to examine separate categories of non-verbal interventions by supplementing the direct evidence with indirect evidence. The network meta-analysis demonstrated a strong effect of yoga therapy on the reduction of depressive symptoms. Interestingly treatment as usual was more effective than occupational therapy, and exercise therapy did not have a significant effect on the reduction of depressive symptomatology. Based on the combination of direct and indirect evidence, yoga therapy appeared most effective. Overall, this meta-analysis supports the evidence that non-verbal therapies are effective at treating depressive symptoms, not only in depressive disorders as was shown in studies by Maratos and colleagues (2008), Pilkington and colleagues (2005), or Tkachuk and Martin (1999) [15–17], but also in psychotic disorders.

Even though guidelines for psychosis refer to the depression guidelines in case of comorbid depression, as of yet no studies have examined the effectiveness of CBT targeted at depressive symptoms in psychosis directly. CBT primarily directed at delusions and hallucinations has a moderate positive effect (Cohen’s *d*: 0.36,) on mood in psychosis [14], yet as a secondary outcome. This effect size is comparable to the effects of CBT on depressive symptoms in depressive disorders (Cohen’s *d*.: 0.42)[59]. According to the current meta-analysis, the effect size of non-verbal therapies on depressive symptoms appears larger than the effectiveness of CBT (non-verbal therapy E.S.: 0.81). However, it is important to keep in mind that the size and qualities of the studies in support of CBTp are considerably higher than in the current review.

A common factor of non-verbal therapies is that they are thought to relieve negative symptoms by using activating strategies [20], such as running, playing an instrument, or dancing. Non-verbal therapies might reduce depressive symptoms in psychosis by stimulating activity, and thus overcoming apathy that is often found in depressions. CBTp on the other hand, is thought to reduce depressive symptoms as a secondary effect, through the relief of positive symptoms [14], and this effect is maintained over a nine months follow-up period [60]. Whether the effect on depressive symptomatology of non-verbal therapies is maintained over a longer period, is unknown.

### Limitations

In the current meta-analysis, only three studies [41,43,45] were of good methodological quality. The other studies are of low to moderate quality, due to a number of reasons such as a lack of (i) a description of the randomization process, (ii) independent assessors, (iii) intention-to-treat analysis, (iv) a sufficient sample size, or (v) appropriate blinding. Given the low number of high quality studies we were not able to conduct a sensitivity analyses. A general suggestion for future research would be for studies to make use of the guidelines of conducting clinical trials as described in the CONSORT statement [61]. Moreover, given that only two studies reported long-term effects, we were not able to draw conclusions about durability of effects.

There was an indication for a publication bias. Inspection of the included studies revealed this is likely due to one potential outlier [42], with an extremely high effect size. Therefore, it should be kept in mind that findings may have been inflated for the non-verbal therapy outcome. As the music therapy outcome and the network analysis did not include the outlier, interpretation of these findings are more straightforward.

To determine effectiveness of a supposed intervention, direct evidence from RCT’s are usually preferred. Due to the heterogeneous nature of the identified direct comparisons, indirect evidence was also added to this meta-analysis. Given that concerns have been raised about the certainty of combining direct and indirect evidence, all outcomes from our network model need not be interpreted as definite proof of effectiveness (or non-effectiveness), but more of an speculative indication as to its potential effectiveness. The direct evidence from this meta-analysis can therefore be viewed as more reliable than the combination of both indirect and direct evidence.

### Clinical Implications and Future Research

Non-verbal therapies overall lead to a reduction of depressive symptoms in psychotic disorders. Specifically music therapy and yoga therapy showed a positive effect on the reduction of depression. Exercise therapy and occupational therapy did not have an effect on depressive symptoms, although this outcome is speculative given the inconsistency of evidence. Overall, the current meta-analysis provides preliminary evidence that non-verbal therapies may be recommended for the treatment of depressive symptoms in patients with psychotic disorders. The interventions included in this review have formats that seem feasible to implement in clinical practice. The number of sessions varied widely across and within the types of non-verbal therapies, ranging from 4 to 48 sessions, over a period of 2 to 24 weeks. No clear relationship was observed between the number of sessions and the effect size. The included studies reported positive effects of yoga and music therapy that were mostly delivered in a group format, whilst exercise therapy was mostly provided on an individual basis. Given the different formats utilized by the studies, and that the effect of the specific formats (number of sessions, group or individual format) have not been investigated, no firm conclusions about an optimal format can be drawn for clinical practice.

There are a number of recommendations for future research. Generally, there is a clear need for more high quality randomized controlled trials investigating the effectiveness of non-verbal therapies for the treatment of depressive symptoms as a primary outcome measure in psychotic disorders. Yoga and exercise therapy studies in particular regularly omit the measurement of depressive symptoms, and focus on general psychotic symptoms instead [62]. Moreover, given only two studies in this meta-analysis carried out follow-up measurements, long-term effects should be addressed in future studies. Currently the durability of non-verbal therapy outcomes remains unclear. Suitable control conditions is another factor which needs to be attended to. For music therapy studies, it is recommended to include an active control condition, which allows the investigation for the specific effect of music therapy. On the other hand, yoga and exercise therapy studies often use active control conditions, which is to be applauded.

A fruitful area for future research is to examine the mechanism of action in non-verbal therapies, as we are currently speculating regarding their active ingredients. For example, stimulating social interactions is believed to be one of the active ingredients in music therapy, yet we cannot be certain that this is what makes music therapy effective. Specifically measuring social interactions/skills in a music therapy study and evaluating its role as a mediator between music therapy, and a decrease in depressive symptoms, would yield more insight into this mechanism. Moreover, given the likely different mechanisms of action in verbal and non-verbal therapies, it would be informative to directly compare these types of therapies in the treatment of depressive symptoms in psychotic disorders. This would allow for the examination of whether non-verbal therapy as compared to verbal therapy is more or less effective at treating depressive symptoms in psychosis specifically.

To conclude, the evidence suggests non-verbal therapies may be effective at treating depressive symptoms in psychosis, yet future research needs to be of higher quality, address long-term effects, and include active control groups. In addition, future studies examining the overall effect of non-verbal therapy in psychotic disorders should aim to include measurements of depression.

## Supporting Information

S1 PRISMA Checklist Prisma Checklist. (DOC)
