# Correction: Treating Depressive Symptoms in Psychosis: A Network Meta-Analysis on the Effects of Non-Verbal Therapies

**DOI:** 10.1371/journal.pone.0209762

**Published:** 2018-12-19

**Authors:** Laura A. Steenhuis, Maaike H. Nauta, Claudi L. H. Bockting, Gerdina H. M. Pijnenborg

The third author's name is spelled incorrectly. The correct name is: Claudi L. H. Bockting. The correct citation is: Steenhuis LA, Nauta MH, Bockting CLH, Pijnenborg GHM (2015) Treating Depressive Symptoms in Psychosis: A Network Meta-Analysis on the Effects of Non-Verbal Therapies. PLoS ONE 10(10): e0140637. https://doi.org/10.1371/journal.pone.0140637
